# Delivery of public health services by community health workers (CHWs) in primary health care settings in China: a systematic review (1996–2016)

**DOI:** 10.1186/s41256-018-0072-0

**Published:** 2018-06-06

**Authors:** Wenting Huang, Hongfei Long, Jiang Li, Sha Tao, Pinpin Zheng, Shenglan Tang, Abu S. Abdullah

**Affiliations:** 1 Global Health Program, Duke Kunshan University, Jiangsu, 215347 China; 2 Department of Preventive Medicine, School of Public Health, Fudan University, Shanghai, 200032 China; 3 Duke Global Health Institute, Duke University, Durham, NC 27710 USA; 4 Boston University School of Medicine, Boston Medical Center, Boston, MA 02118 USA

**Keywords:** Community health worker, CHW, Village doctor, Primary health care, China

## Abstract

**Background:**

Community Health Workers (CHWs) have been widely used in response to the shortage of skilled health workers especially in resource limited areas. China has a long history of involving CHWs in public health intervention project. CHWs in China called village doctors who have both treatment and public health responsibilities. This systematic review aimed to identify the types of public health services provided by CHWs and summarized potential barriers and facilitating factors in the delivery of these services.

**Methods:**

We searched studies published in Chinese or English, on Medline, PubMed, Cochrane, Google Scholar, and CNKI for public health services delivered by CHWs in China, during 1996–2016. The role of CHWs, training for CHWs, challenges, and facilitating factors were extracted from reviewed studies.

**Results:**

Guided by National Basic Public Health Service Standards, services provided by CHW covered five major areas of noncommunicable diseases (NCDs) including diabetes and/or hypertension, cancer, mental health, cardiovascular diseases, and common NCD risk factors, as well as general services including reproductive health, tuberculosis, child health, vaccination, and other services. Not many studies investigated the barriers and facilitating factors of their programs, and none reported cost-effectiveness of the intervention. Barriers challenging the sustainability of the CHWs led projects were transportation, nature of official support, quantity and quality of CHWs, training of CHWs, incentives for CHWs, and maintaining a good rapport between CHWs and target population. Facilitating factors included positive official support, integration with the existing health system, financial support, considering CHW’s perspectives, and technology support.

**Conclusion:**

CHWs appear to frequently engage in implementing diverse public health intervention programs in China. Facilitators and barriers identified are comparable to those identified in high income countries. Future CHWs-led programs should consider incorporating the common barriers and facilitators identified in the current study to maximize the benefits of these programs.

## Background

The World Health Organization (WHO) has identified the global chronic shortage of skilled health workers in the *World Health Report* [1]. This shortfall of available skilled health workers has been estimated to be as high as 4.25 million in Africa and Asia [1]. The quality and density of human resources for health has been widely considered as one of the main contributors of maternal and child health outcomes and other health inequalities [2, 3]. In the attempt to deal with this health workers crisis, many countries, especially low- and middle-income countries (LMICs) have widely used community health workers (CHWs) to support the underserved population in resource-limited settings and deliver key health care and health promotion interventions in their communities [4].

According to WHO, CHWs consist of different community health aides, but not trained health professionals, who are selected and trained to work in their own communities [1]. They are usually trained to deliver various basic and health-related interventions and services within their own community. However, CHWs may have different titles because their specific job responsibilities within their local cultures and health systems vary (e.g., traditional birth attendant, community health volunteer, village health worker, village doctors, health advocates etc.). It is difficult to generalize one universal title for all CHWs [1]. We will use the term “CHWs” to describe all these categories of healthcare workers in this paper, unless specified otherwise.

Evidence from various countries has shown that CHWs are able to make effective contributions in health outcome, particularly in maternal and child health [5–7]. One of the best-known programs of CHWs is the “barefoot doctors” which was implemented in China from the 1950s to the early 1980s. Around one million agricultural workers were trained to be the “barefoot doctors” to provide primary health care, first aid, and health education [8]. They significantly improved rural health care coverage and infectious disease control and dramatically reduced the national infant mortality rate [9]. However, in 1981, as the national health system shifted from a cooperative medical system to a private medical system, the barefoot doctor program was abolished [10]. In this private medical system, the “barefoot doctors” still served as frontline healthcare workforce in primary health care level. Their title became “village doctors” if they passed the national exam of the village doctor, or their title became “village health aides” if they failed.

**Fig. 1:**
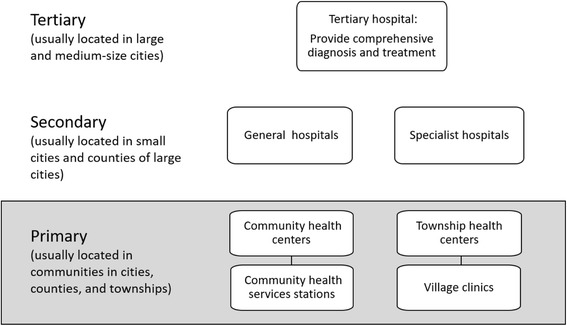
Structure of the Chinese Health System

Currently, China’s health system consists of three levels: tertiary, secondary and primary levels (Fig. 1). Tertiary hospitals are responsible for the majority of comprehensive diagnosis and treatment. They have full coverage of diverse medical and surgical departments and are equipped with modern medical and diagnostic equipment. These hospitals exist in large and medium-size cities. Secondary hospitals include general hospitals in small cities and counties of large cities, as well as most specialist hospitals. However, the CHWs only served in primary health care level. Primary health service is provided by medical institutions, which refers to basic level health service institutions in residential areas in urban or rural town health centers. The scale of community health centers varies greatly. In large cities like Shanghai and Beijing, community health centers (CHCs) are developed from some small secondary hospitals with inpatient care. The number of CHWs in each CHC may vary between 5 and 10). Other primary health centers, however, especially health stations in rural areas, only have a limited number of doctors (varies between 1 and 5), the so-called village doctors, to provide basic consultancy services. Generally, the population that each CHW serves ranges from 300 to 2500 residents [2].

In recent years, the Chinese Ministry of Health has started to emphasize more to improve the primary health care services by incorporating the community based services within the primary healthcare system [11, 12]. Since then the function of basic medical service and public health services has been integrated into primary healthcare level (community health services centers). In accordance with the provisions of the National Basic Public Health Service Standards, issued by the Ministry of Health in 2011, community-based health services should include the following aspects: health records, health education, immunization, infant care, maternal care, health management of elderly, health management of patient with hypertension, type II diabetes, mental illness and infectious disease, as well as public health emergencies report and treatment [11, 12]. All of these services are delivered by existing healthcare personnel working in community health centers including CHWs; usually Chinese traditional medicine services are not provided in the community health centers.

Although the village doctors provide both treatment and public health services, they usually focus more on treatment instead of public health service, due to the inadequate financial incentives to deliver public health service and heavy workload [13]. Besides financial incentives, studies in other countries provided evidence that CHWs performances can be affected by recruitment process, workload, and retention policies [14–16]. Policies on incentives, career perspectives, and supervision have great influence on CHW’s motivation. In addition, reviews also showed that basic and continuing training and education can enhance CHW’s performance [17–19]. However, limited studies were conducted on this frontline workforce of primary healthcare provider in China. Understanding the pattern of services provided by CHWs and the challenges and barriers faced by CHWs will guide the policy makers in assessing the potential to integrate CHWs within primary health care delivery systems. Also, to address shortages of healthcare workforce, many developing countries are now examining the potential to engage CHWs to deliver primary healthcare services. Experiences from China would be useful to guide these countries in developing local policy strategies to integrate CHWs within primary health care delivery systems. Therefore, we conducted a systematic review of intervention studies involving CHWs, to identify the types of public health services provided by CHWs and summarized potential barriers and facilitating factors in the delivery of these services. This systematic review will be guided by the following research question: What are the types of public health programs provided by CHWs in China as reported in studies from 1996 to 2006, and what are the barriers and facilitating factors?

## Method

### Search strategy and procedure

We conducted a systematic review of all manuscripts published in peer-reviewed English and Chinese language journals about the topic of the role of CHWs in primary health service delivery in China. Following a protocol, the literature review began with a search on PubMed, Cochrane, Google Scholar, and CNKI (China National Knowledge Infrastructure, China Academic Journals full-text database) using two combinations of the medical subject heading (MeSH): ‘community health worker’ and ‘China’; ‘village doctor’ and ‘China’. After identifying initial studies, the additional keywords, ‘midwifery’, ‘reproductive health’, ‘family planning’, and Non-communicable diseases like ‘hypertension’, ‘diabetes’, ‘mental’, ‘chronic’, ‘cardiovascular diseases (CVDs)’, ‘stroke’, ‘cancer’, ‘chronic obstructive pulmonary diseases (COPDs)’, ‘physical activity’, ‘obesity’, ‘diet’, ‘tobacco’, ‘smoking’, ‘alcohol’ were used combine with the initial keywords. These keywords were also translated into Chinese when searching Chinese literatures via CNKI. We restricted our review to the manuscripts that were published in the last 20 years (1996–2016). We used the publication date instead of the study date for consistency since publication date is more accessible than the study date. We also used the link to related articles in PubMed and CNKI for initially selected articles. After searching the manuscripts with keywords, the reference lists of these manuscripts were hand-searched to identify additional publications.

Each manuscript was assigned a reference number. Each manuscript includes the title, types of program, terms used to define CHWs, the role of CHWs, program duration, type of training delivered to CHWs, challenges, and facilitating factors.

### Inclusion and exclusion criteria

The inclusion criteria for CHWs-delivered studies included:

1. *Participants*: Participants can be patients or the general population. We do not have specific requirements for participants since various people could be the receiver of these public health services.
2. *Intervention types*: Preventive measures or health promotion interventions that were provided by CHWs.
3. *Comparison*: Not applicable.
4. *Outcome*: Delivery of reported intervention.
5. *Study types*: Intervention studies conducted in China which focused on public health services including health education, reproductive health and family planning, managing patients with infectious disease, child health, vaccination, and common NCDs (i.e., hypertension, diabetes, cardiovascular disease, cancer and mental health.

The exclusion criteria included: i) articles that did not focus on China; ii) articles that focused on the health professionals (physicians, doctors, nurses) rather than CHWS as we have defined for this review; and iii) articles that did not describe structured public health interventions (e.g., news, conference reports, books, reviews, health system analysis, disease prevalence).

### Data extraction

Using the above inclusion and exclusion criteria, two reviewers (WH and HL) identified relevant studies independently. Each reviewer screened the titles and abstracts of the potential articles to assess their eligibility for this review. When there was disagreement, the decision to include a study was made after discussion and consensus by both reviewers and, in some cases with input from the project leader (ASA). We then read the full texts of all eligible materials and summarized relevant content. Using an Excel form, we assigned each eligible article with a unique reference number and extracted the following information: the types of program, titles of CHWs, the services provided by or/and the responsibilities of CHWs, program duration, training received by CHWs, challenges and facilitators faced by CHWs in the engaged program. We also summarized the training types received by CHWs and the training duration respectively, if this information was available.

## Results

### General description

**Fig. 2:**
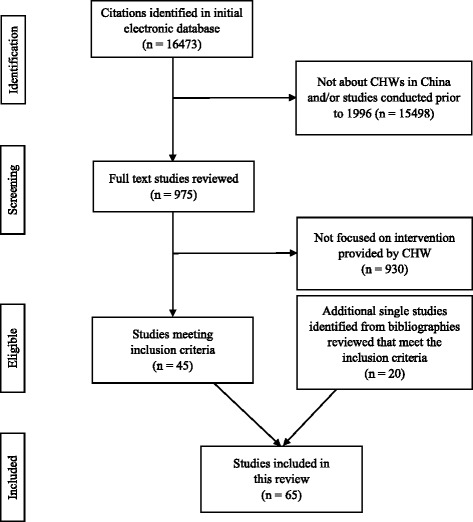
Selection Process for Identifying Relevant Studies

We identified 65 full-text published studies; 43 in English (Table 1) and 22 in Chinese (Table 2) that fit our criteria from 16,473 articles. In Fig. 2 we described the article screening process that followed PRISMA flow diagram [20]. Only one study evaluated a nationwide program [21]. Fifty-one studies (22 in Chinese) were single site studies conducted evenly in east, central, and west China. Thirteen studies (all English) were the multi-site programs that ranged from two to eight sites [22–25].

**Table 1:** Description of Health Intervention Program Involving Community Health Workers (CHWs) in English literature (*n* = 43)

Author	Year Location	Names of CHWs	Program Duration	The Role of CHWs	Types of Training	Challenges (−)	Facilitating factors (+)
Reproductive Health							
Levi A, Factor D, and Deutsch K [41].	2013 Yushu, Qinghai province	Community Health Workers (CHWs)	6 years	Health education (women empowerment), basic maternal care, referral, conduct prenatal visits, identify danger signs, attend births and visit newborn	Basic knowledge, referral, conduct prenatal visits, identify danger signs, attend births and visit newborn	1. Program sustainability; 2. Various quality of the CHWs training	1. Strategic planning; 2. Government support; 3. Clinic support
Jiang, et al. [77]	2016 Guangxi province	Traditional Birth Attendance (TBAs); Village Maternal Health Care Workers	Not reported	Mobilization of pregnant women for institutional delivery, assisting with home visit for basic care and escorting pregnant women to the hospital for childbirth.	Different levels of training in Maternal Child Health hospitals: emphasized identifying high-risk pregnancies and assisting with referrals; for TBAs, focused on care during childbirth and referral skills; for trained birth attendance (TrBAs), additional midwifery training and were required to conduct at least 30 independent deliveries under the supervision of an obstetrician.	1. How to deal with TBAs; 2. Logistical challenge of institution-based delivery in remote areas.	1. Sufficient and comprehensive preparation within the health system, including training of health human resources, building infrastructure, improvement of service quality, and establishment of referral channels and quality referral centers. 2. Financial support from county hospitals or township health centers.
Dickerson, et al. [76]	2010 Tibet	Outreach Provider (both local healthcare worker and laypersons)	20 months	Maternal-newborn education including antepartum/postpartum care seeking and nutrition; birth planning and maternal newborn danger sign recognition; skilled attendance at birth; clean delivery practices; prevention of postpartum hemorrhage (PPH), birth asphyxia, and neonatal hypothermia and hypoglycemia; proper care of the umbilical cord; and breast-feeding and postnatal care seeking.	Training contend focus on maternal-new born health education, hands-on skills, material resources distribution. Role-playing is the most common learning method.	Not reported	Not reported
Tu, et al. [25]	2004 Eight Chinese sites: Shanghai and Chongqing cities and Hebei, Henan, Jiangsu, Zhejiang, Fujian, and Sichuan province	Family-planning workers, including contraceptive providers and community-based distributors.	Since 1970s.	Contraceptive providers are in charge of providing contraceptives to the local family-planning service units at the primary community level and managing and supervising contraceptives. Community-based distributors are in charge of distributing contraceptives and providing general counselling for clients in their service areas.	Not reported	1. Family-planning providers were ambivalent about the provision of sexual and reproductive health services to unmarried young people. 2. Continued adherence to traditional norms, ambiguities and limitations in the current policy. 3. The family planning workers’ recognition of the need to protect the sexual health of unmarried young people.	1. Family-planning workers are clearly concerned for the well-being of unmarried young people 2. They agreed with the establishment of programmes that improving unmarried young people’s knowledge of sexual and reproductive health. 3. They seemed willing to empower the government to establish educational and service delivery programmes for unmarried young people.
Tang, et al. [79]	2009 Yunnan province	Village Doctor (VD), family planning workers, women’s cadres, and teachers	28 months	Reproductive health knowledge education that based on Internet: family planning and safe practice, maternal and child health RTI/STI/HIV prevention and control, adolescent sexual health, gender consciousness, development of women’s identity, health promotion and health education	Computer skills training workshop	1. There was no recertification mechanism to motivate village doctors to upgrade their knowledge and skills and to improve practice.	1. Using the website as one of the main strategies to improve village doctors’ knowledge, attitudes, and practices and to close the distance between urban and rural areas.
Edwards & Roelofs [42]	2006 Yunnan province	Grassroots maternal and child health worker; VD; traditional village midwives	6 years:	Not reported	Holistic learning methodology (skills in communication and group dynamics, critical analysis, clinical skills, and personal growth); participatory training with methods centred on cycles of reflection-action-assessment; supportive working relationships fostered among different categories of health workers at village, township, county, and provincial levels.	1. Doubts from work unit leaders; 2. Various learning needs; 3. Different literacy levels; 4. Unequal clinical competencies	1. Strong, transparent partnerships (deep engagement with local partners); 2. Official support from government; 3. Maintaining a good fit between core project elements and the existing health system; 4. Creating supporting organizational structures; 5. Designing a transition plan at the start of the project
Zeng, et al. [80]	2008 Shaanxi province	VD	3.5 years	Conduct mini-survey of all women of reproductive age at the beginning; Recruit participants; obtain informed consent; visit participants every two weeks to provide more supplements and to retrieve the used blister strips and record the number of remaining capsules.	has training for VD, but did not mention the content of training	Not reported	Not reported
Ma, et al. [27]	2010 Shen County in the central China	Village nurse	2 months	Recruitment and distribution of the supplements, home visit once a week, provide counselling about the possible side effects	Not reported	Not reported	Not reported
Sun, et al. [80]	2010 Shen County in the central China	Village nurse	2 months	Home visit once a week, replenish supplements and monitor compliance by counting and recording the number of supplements that were taken	Not reported	Not reported	Not reported
Hemminki, et al. [23]	2013 Anhui province, Shanxi province, Chongqing city	VD and family planning worker	2 years	Provide health education and encourage pregnant women to seek health care; inform township health centers of pregnancies in their villages; postnatal care through phone consultation or home visits.	Health education communication skills was provided to both township midwives, village doctors and village family planning workers. Lectures covered maternal health care regulations and self-care during pregnancy and recognition of risk during pregnancy. Group discussions and role-plays.	1. In the training, teachers may not have known how the midwives worked or what situation and problem they faced in their work. 2. Modern teaching methods like small-group were not feasible because of too many trainees. 3. Some VD do not want to do health education due to lack of financial compensation.	Not reported
Tuberculosis							
Tao, et al. [24]	2013 Qinghai province, Hebei provinc, Henan province, Jiangsu province	VD	Not reported	Directly observe every dosing of smearing positive TB patients during the whole treatment period either on facility-based or home-based. A family member can be accepted as DOT provider after training for those families living in extremely remote areas.	No detail information about the training content.	1. DOT allowance did not reach to the doctors; 2. Lack of a performance-based incentive approach; 3. Inconvenient transportation system; 4. Shortage of hands, time conflict between DOT and routine jobs; 5. Insufficient capacity of village doctors on home-based DOT; 6. TB stigma; 7. Low effect of training programs 8. Lack of subsidies	1. Raising both monetary and non-monetary incentives of DOT rural health workers
Gai, et al. [82]	2008 Shandong province	VD	Since 1990s	Education program for patients and rural residents, including distribution of pamphlets, verbal announcements, village broadcasts, and bulletins. Case detection and supervised patients.	Occupational training in TB control and treatment.	1. Village doctors are recognized their current knowledge was insufficient to meet the demands of their work. 2. Some practices of village doctors were inappropriate for patient referral	Not reported
Wei, et al. [82]	2008 Guangxi province and Shandong province	VDs; family member	1 year	Diagnosis, prepare TB treatment, follow up, and determine treatment outcomes. Follow up: Select a family members as their treatment supporter and train them in this role (intervention group)/observe the patient taking drugs (control group)	1) Introduction of the desk guide and how to use a guideline in practice; 2) Strengthening communication between doctors and TB patients; 3) Educating patients and choosing a treatment supporter; 4) Educating the TB supporter; 5) Reviewing patients at the county TB dispensary.	1. Economic development and road accessibility	1. Giving local policy-makers and practitioners a lead while making changes in policy and practice. 2. Systematic approach to adaptation and scale-up. 3. The adapted guideline and other materials were replicable and sustainable for scale-up.
Sun, et al. [80]	2008 Shandong Province	VD	Since 1990s	Monitor the patients taking their medications at the right time at the right dose.	Not reported	Not reported	Not reported
Xiong, et al. [83]	2007 Hubei province	VD	1 year evaluation	Survey, trace and refer suspects (patients with TB symptoms) to county TB dispensaries or other designated sputum examination centres.	1. Technical training (the provincial workgroup drew up a strategic plan and trained TB staff from 30 county TB dispensaries. 2. A total of 35,000 desk calendars with information about TB and control policy were printed and delivered to village doctors, patients and village leaders.	1. Main reasons of the low follow-up rate were the shortages of funds and human resources. 2. A mobile population and inaccurate information were the main causes of the low follow-up success rate.	Not reported
China Tuberculosis Control Collaboration [21]	1996 Nationwide	VD	Started at 1991	Observing every dose of the TB drug; follow up the patient who do not come for their treatment.	Not reported	Not reported	1. Top-down approach; 2. Supervision of staff was facilitated by system of record-keeping that is easily understand but difficult to falsify, including separate district registers, laboratory registers, and treatment cards.
Meng, et al. [79]	2004 Shandong province	VD	Started at 1992	Observing every dose of the TB drug	Not reported	1. VDs were not willing to provide this kind of services because of no financial incentives; 2. TB health experts thought that drug talking without supervision by the VDs was acceptable; 3. TB patients may find it inconvenient to go to a village clinic to take the drugs	Not reported
Tobacco Control							
Abdullah, et al. [62]	2015 Shanghai city	CHWs	6 months	Intervention including 6 individualized counseling sessions about children second-hand smoke exposure.	Practicum training, including lectures, in-class discussion, case reviews, and role-plays	1. Maintain the communication between participants and CHWs	1. The satisfaction with CHWs
Child Health and Vaccination							
Jin, Sun, Jiang and Shen [61]	2005 Hefei city, Anhui Province	VD	Around 6 months	Early childhood development consulting	Training is based on the WHO’s teaching materials about the technique of early child healthcare, using reading, videotape presenting, and practice to improve the knowledge and ability of village doctors.	1. Village doctors were unwilling to conduct the consultation because there was no additional financial reward.	1. Mothers were eager to learn more about early childhood development and willing to practice and apply it.
Wang, et al. [71]	2007 Hunan province	Village-based Health Workers	1 year	Administer using auto-disable syringe and administer vaccine storage for hepatitis B.	Not reported	Not reported	Not reported
Chen, et al. [22]	2016 Xuanhua city, Sichuan province	VD	Not reported	Use the app to make appointment, record, and track children’s immunization status, to remind the caregiver about immunization	The use of EPI app	1. Only include younger ones, older village doctors may be limited; migrant children; 2. Caregivers changed their cell phone numbers	1. mHealth technology is helpful.
NCD related - Diabetes and/or Hypertension							
Feng et al. [43]	2013 Lu’An city, An’hui province	VD	6 years (every 12 months for plasma glucose and ever month for body weight and blood pressure)	Conduct glucose screening; measuring body weight and blood pressure; provide counseling on glucose screening; promote screening participation (during each biannual follow up glucose screening); referral; provide behavior change counseling for pre-diabetics	Web-based training and A comprehensive ‘occupational toolkit’ consists of a workbook, a manual and a set of cue-cards, providing knowledges on diabetes and working guidance to assist the VDs’ practice. E.g., Each cue-card enlists critical steps or elements for delivering a specific type of counseling; the manual is a reference book including elementary protocols (e.g., diabetes screening performance, dietary modification counseling, etc), common problems and solution tips, and fundamentals of diabetes prevention (e.g., basic knowledge for intervention execution)	1.Most village doctors are currently unaware of and certainly not practicing in diabetes prevention; 2. Heavy workload already; 3. The project heavily relies on electronic support, the actual practice may beyond the ability of VDs’ and elder villagers’ in rural area to use computerized systems	1. Trust from the patient and communities; 2. The service itself is not complex, capable for VDs (only 15 min); 3. Well-established guidelines and manuals; 4. Village clinics provide appropriate settings for diabetes measurement and counseling; 5. Electronic support and web-based training are cost-saving and time flexible; and it allows continuous expansion of trainees; 6. Performance-based incentives; 7. Local health authorities support on resources
Lin et al. [44]	2014 Xilingol county; Inner Mongolia	VD	4 years	Case management and monitoring via Electronic Health Record; follow-up via regular visits, measure blood pressure and blood sugar levels; check medication compliance	Not reported	1. Lack of policy support from the health system	1. Closely connect with higher levels of the healthcare system and benefit the rural area, if implemented in large-scale
Chen et al. [36]	2014 Lu An; Anhui province	VD	6 months (1 month per session)	Identifying high-risk patients, and follow-up counseling on lifestyle modification, health education on diabetes risk, balanced diet, and physical activity	Instructions on the application method of the program, with standardized “step-by-step” navigation for VDs to follow in practice	1. Lack of electricity security (facility) in remote settings; 2. Communication difficulties: sometimes unable to engage patients in completing every listed item in the instruction.	1. Innovative; 2. Easy to follow the navigation; Professional knowledge built in the program helps in the case identification and management; 3. High acceptance rate among diabetes patient.
Zhong et al. [56]	2015 Tonglin, Hefei province, Bangbu, Anhui province	Peer Leaders; Community Health Service Center (CHSC) Staff	6 months /session	Biweekly educational meetings Co-led by peer leaders (PL) and staff of Community Health Service Centers (CHSCs). Topics: diet, physical activity, medications, foot care, stress management. PL: outreach, promotion, emotional support meeting and non-professional activity (Tai Ji, morning exercise, etc.)	Not reported	1. Lack of staff resources in some sub-communities (organizational support from hospitals)	1. Close relationship with peer leaders; 2. Knowledge; 3. High patient engagement
Li et al. [102]	2015 3 provinces in China, specific location was not mentioned	VD	(cross-sectional survey among VDs)	Providing hypertension and/or diabetes case management; create citizen health record	Routine training programs including content like health care policy, standards, basic public health services (BPHS) quality management, and the norms, standards and service delivery paths of BPHS.	1. Limited compensation, low financial incentive, uneven geographic coverage of the New Cooperative Medical Scheme insurance contract	1. More education, more training opportunities, receiving more public health care subsidy; 2. Integrated management and supervision; 3. Being a New Cooperative Medical Scheme insurance program-contracted provide
Browning et al. [54]	2016 Fengtai District, Beijing	Health coach (health workers from the local community health station (CHS))	1 year	Conduct bi-weekly/monthly telephone and face-to-face motivational interview (MI) health coaching as psychosocial supporting and lifestyle counseling approaches to improve the outcome of glycemic control and self-care of T2DM patients.	Key concepts in patient-centred communications, health psychology, epidemiology of key targeted illnesses and conditions, the framework and rationale of MI, and the application of MI core skills across the behavior change process. Review workshop of these techniques will be arranged at 1 month after the project initiate, and every 3 months after that.	1. Long-term effectiveness needs to be assessed; 2. Not generalizable to rural settings with few human resource	1. Good learning and practice capacity; 2. Well-organized training process including review workshops; 3. Pilot study - quality control
Peiris et al. [55]	2016 Beijing; Hebei province	Lay Family Health Promoters (FHP); Healthcare staff	2 years	Healthcare staffs: case monitoring, provide support to FHPs via communication tools built inside the SMARTDiabetes application; FHPs: report the progress and update EHR data on behalf of the patients (i.e. Their families who have diabetes) via the SMARTDiabetes application. Co-determine action plan with the support from healthcare staffs. Experience sharing with other FHPs in the community via App-based forum.	Installation and the use of the technology and management of diabetes	1. Hard to generalize for other contexts without electronic health record infrastructure, and for the population with limited access to smartphone technology	1. Cost-saving; 2. Time-saving; 3. Strong motivation of FHPs to support families with diabetes; 4. Close communication between clinical healthcare staffs and FHPs
NCD related - Cancer							
Belinson et al. [45]	2014 Henan Province	Community Leaders (CLs); promoters; local health worker	3 years	Joint tasks for CLs and promoters: gather personal information; label the specimens and follow the procedures; advertisement and community notification about the screening program via video, posters, workshops. CLs: instruct sample collection; Local health workers: consulting after results generated, refer positives to visit clinics for management.	Meaning of a positive test; Management options and techniques; via video and workshops	Not reported	1. Good communication skills; 2. Enthusiasm for the community-based screening model; 3. Community, institutional and government support
Chai et al. [46]	2015 An’hui province	VD	5 years	1. Provide health counseling regarding: alerting risks and harms; setting objective behaviors; discussing efficacy and benefits; anticipating barriers and problems; 2. Risk assessment promotion; 3. Providing assistance and supports on healthy lifestyle; assist and support patients’ behavioral change (reviewing behavior changes, encouraging improvement, identify and select problems, and solve problems); 4. Manage, record and post typical cases bimonthly on a web forum and share experiences with other experts and VDs (prevention and management)	Web-based tutorial on implementing the project prevention in both video and textual formats; typical case studies as references for practice; video and pictorial materials about cancer and its prevention	1.The project heavily relies on electronic support, the actual practice may beyond the ability of VDs’ in remote rural area to use computerized systems	1. Performance-based incentive and awards; 2. Well-established web-based support and supervision system are technically helpful and time-saving for VDs to practice; 3. The user-friendly education and learning assistance; 4. Self-practice, encouragement, and problem inquiring and answering allow most village doctors became confident users of the electronic support system
NCD related - Mental Health							
Prince et al. [60]	2007 urban and rural catchment, no specific location mentioned	CHWs	2 years	Help the researchers to detect high-risk population, being the community key informants of the research team	Not reported	Not reported	Not reported
Gong et al. [61]	2014 Liuyang city, Hunan province	VD	1 year	1. Develop and maintain case files for every schizophrenia patient. 2. Store and distribute antipsychotics to family members on a weekly basis, or directly observe drug-taking (DOT) at the village clinic on a daily basis. 3. Accompany patients and family members on bimonthly visits to psychiatrists for drug dispensation in order to participate in assessing patients’ mental status and explain treatment plans to patients and their families. 4. Record patients’ medication-taking behavior weekly. 5. Identify signs of relapse in order to provide prompt referral services.	Mental health knowledge, case-management skills, and directly observed therapy (DOT).	1. Overload already, no time for extra work; 2. Chinese healthcare system does not compensate VDs financially for extra effort in providing mental health services; 3. Inadequate engagement from patients and patient’s family	1. Under the national “686” mental health scheme - government support; 2. Consistent collaboration with local government; 3. Training protocol met with local VDs’ competence and expectations
Chen et al. [62]	2014 Xuhui and Hongkou Districts; Shanghai	CHWs	2 years	Work with community psychiatrist and nurse as a team to conduct case management: 1. assess the health condition, recovery status, daily functioning, employment status, and social activities of participants; 2. assess patients’ needs to provide references for developing personalized rehabilitation plan; 3. develop personalized rehabilitation plan and assist the patient to cope with the plan: drug adherence training, daily skills training, family psychological intervention; 4. monthly individual follow-up to refine the intervention plan; 5. participate the already established training course	Not reported	Not reported	Not reported
Zhou and Gu. [63]	2014 Shanghai	CHWs	2.5 years	Assist chronic schizophrenia patients with self-management. After each patient received weekly self-management skill training, CHWs reviewed patients’ self-management checklist (record their daily adherence quality of sleep, occurrence of side effects, occurrence of residual symptoms and early signs of relapse, daily activities, and general mood) every month on a group meeting to supervise the adherence and collect records	Not reported	Not reported	Not reported
Ma et al. [63]	2015 Guangxi province	Primary health care providers	2006-now	Community education, medication distribution; observe compliance and life status; report side effects or any abnormality; referral and follow-up	Training provided by the national ‘686 project’: mental health disease management, education and social treatment and prevention of mental illness	1. Lack of professional knowledge; 2. Fear of patients’ attack; 3. More extra work; 4. No management approach 5. Less subsidies	1. The capacity to use communication skills with patients and their family members, have proper attitude (without discrimination); 2. Understand the professional knowledge of mental health 3. More income/subsidy
Tang et al. [52]	2015 Mianzhu, Sichuan province	VD	2 months	Conduct weekly intervention with elderly depression patients using cognitive behavioral therapy techniques to: 1.do physical examination; 2. identify emotion status and negative automatic thoughts; 3. proceed psychological intervention; 4. provide problem solving method	Workshop on mental disorder knowledge, counseling concepts and techniques, with specific focus on cognitive behavioral therapy. Practice through role-play. Trainings were conducted by one qualified cognitive therapist	1. Time constraint for training; 2. Under-developed training manuals and the inadequate practice, caused anxiety and a sense of incompetence; 3. Poor patient adherence - prefer medicine over CBT; 4. No financial incentive	1. Well designed (easy to understand the content) and organized (the use of role play) training; 2. Strong learning ability and interest; already have some relevant knowledge; 3. Local community trust; 4. Multi-disciplinary team
Xu et al. [64]	2016 Liuyang, Hunan Province	VD; Lay health supporters(LHS): mostly family members of the patients	1 year	VD: 1) screening, as the “686” scheme requires; 2) report relapse signs and side effects (based on the texts from LHS) to psychiatrics; 3) team up with LHS, MHA and psychiatrists to assist urgent care. LHS: 1) facilitate patient medication adherence with prompts from the e-reminders; 2) monitor for early signs of relapse and side effects using checklists from the e-monitor and report to VDs; and 3) team up with the VD and the township Mental Health Administrators (MHA) to facilitate treatment adjustments and urgent care	The built-in e-educator mHealth program will send periodic SMS messages to the patient, LHS, MHA and VDs to educate them on schizophrenia symptoms, medication, adherence strategies, relapse, rehabilitation and social resources	1. Local psychiatrists with limited training may deliver inappropriate services; 2. No sustainable funding;	1. Under the national “686” mental health scheme - government support; 2. Full individual and community engagement (mental health administrators, psychiatrists, VDs, patients and their families (i.e. LHS)); 3. mhealth applications as a user-friendly health system strengthening tool in doctor-patient coordination; VD: no additional workload; LHS: care and love for their families (i.e. patients) = the major job motivation; non-monetary award system
NCD related - Cardiovascular diseases							
Ajay et al. [66]	2014 Gongbujiangda county, Linzhou county, Tibet Province	CHWs	1 year	With the smartphone-based electronic decision support, CHWs provide monthly follow-up care; identify high-risk patients; referral; provide therapeutic lifestyle advices (smoking cessation and salt reduction); prescribe two drugs (blood pressure lowering drugs and aspirin)	Training on the intervention protocol, including education on targeted CVD lifestyle risk factors and medications being utilized.	1. Lack of economic and healthcare resources	1. Design of the intervention adapt to local context and culture; 2. Supportive national guidelines and policies on CVD prevention and control
Yan et al. [67]	2014 Hebei, Liaoning, Ningxia, Shanxi and Shaanxi	VD	2 years	1. Identify high-risk individuals by screening all patients who visit the village clinics for any reason; 2. Contact patients with existing diseases or potentially at high risk based on their previous knowledge of the patients to maximize screening; 3. Measure blood pressure, provide lifestyle modification advice and monitor acute symptoms or early signs of clinical events on monthly follow-up with high-risk individuals; 4. Timely referral	A technical package developed to guide village doctors on how to screen, identify, treat, follow up and refer cardiovascular high-risk individuals during their routine services.	Not reported	1. Performance-based feedback and financial incentive payment increased VDs’ motivation of participating in CVD preventive services; 2. Interventions are designed to fit CVD management in resource-limited areas
Tian et al. [66]	2015 Gongbujiangda county, Linzhou county, Tibet Province	CHWs	1 year	With the smartphone-based electronic decision support, CHWs provide monthly follow-up care; identify high-risk patients; referral; provide therapeutic lifestyle advices (smoking cessation and salt reduction); prescribe two drugs (blood pressure lowering drugs and aspirin); screening for new symptoms, diseases, and side effects since the last visit, measuring blood pressure, providing lifestyle counseling,	Training on the intervention protocol, including education on targeted CVD lifestyle risk factors and medications being utilized.	1. The duration of the intervention is too short to observe significant health behavioral change; 2. Lack of economic and healthcare resources in the remote areas	1. Performance-based incentive; 2. Culturally adaptive (lifestyle health education materials are in Tibetan language with culture-specific images); 3. The mobile health technology simplified the intervention process, provided appropriate guidance/data and saved time
NCD related Health Education							
Li et al. [70]	2016 Hebei, Liaoning, Shanxi and Shaanxi provinces; Ningxia	VD	18 months	Work with township health educators to provide health education in forms of public lectures, distribute promotional materials, interactive education sessions with vascular high-risk population, promote salt substitute	Not reported	Not reported	Not reported
Others (Shallow anterior chamber screening and verbal autopsy)							
Nuriyah, et al. [73]	2010 Beijing	CHWs; non-professional health worker	Not reported	Screening of shallow anterior chamber with oblique flashlight test.	Not reported	Not reported	Not reported
Zhang, et al. [103]	2016 Hebei province	VD	Not reported	Conduct verbal autopsy in rural areas.	VA method to become qualified interviewers	1. VD who are older or not familiar with technology may require multiple trainings.	1. Mobile phone-based shortened VA

**Table 2:** Description of Health Intervention Program Involving Community Health Workers (CHWs) in Chinese literature (*n* = 23)

Author	Year Location	Names of CHWs	Program Duration	The Role of CHWs	Types of Training	Challenges (−)	Facilitating factors (+)
Health Education							
Baoan Li [47]	2007 Henan, Anyang	VD	5 years	Provided health educations on healthy lifestyle using black broad, banners, and brochures. The health education included salt reduction, healthy diet, weight control, less alcohol, and smoking cessation.	Government, county CDC provided regular training for VDs on NCDs prevention and control.	VDs lack of knowledges on NCDs prevention, risk factors for NCDs, and principles of NCD treatments.	1. Health education is a cost-effective strategy for preventing NCDs. 2. The intervention program can improve the disease prevention capability of VDs thus emphasize the role of VDs in NCD prevention in rural communities.
Reproductive Health							
Cuilan Guo, et al. [78]	2011 Not reported	CHW	2 years	1. Establishing women’s health care promoting medical team and counseling clinic; 2. Carrying out free medical examination for women; 3. Giving out regular lectures about women’s health; 4. Distributing health education materials; 5. Collecting women’s health issues, health need, and health status through door to door visits; 6. Providing tailored health care and education.	Training to familiar with their responsibility and understanding the purpose and significance of health education and nursing promotion. All team members have to pass the specific exam before implementing the intervention.	1. Most women in the community had a low educational level and lack knowledge on women’s health	1. Policy support on involving all stakeholders in promoting women’s health; 2. Providing special services to elderly women, which could be a high-risk population for hypertension, diabetes, CVD, and cancer.
Su Qian, et al. [81]	2010 Jiangsu Province	Grass-roots women health education, promotion female VDs, and family planning staff	11 months	1. Launching the intervention campaign; 2. Establishing and improving the community women’s health management files 3. Building a platform for exchanging information among medical staffs and women in the community; 4. Providing special services and expanding their health care services for women	All team members were trained before the campaign start. The training content includes the purpose and significance of establishing health education team; specify their roles, tasks, etc.	1. Most women lack basic knowledge on health in the community, including sexual infectious disease, HIV, and intimate partner violence. 2. Other stakeholders need to be involved, including health department, family planning department, civil administration, All-China women’s federation, and administration of justice. 3. Reproductive health education for women needs to be evaluated in the pay-for-performance system for relevant government departments.	Not reported
Yang Haixia, et al. [85]	2008 Yunnan Province	VD	1 year	1. Implementing health education activities: handing out health education manuals, training, etc.; 2. Selecting, educating, and assessing pregnant women’s companion	Not reported	1. The educational level of rural pregnant women were low; 2. The responsibility of VD needs to be strengthen.	1. Adopting peer education (companion for pregnant women) approach which is suitable for rural population; 2. Choosing relatives as companions, usually husband or mother-in-law;
Infectious Disease Control and Prevention							
Lin Wang et al. [78]	2011 Henan Province	VD	6 months	1. Distributing medication of ART to people living with HIV/AIDS (PLWHA) and managing PLWHA. 2. Collecting sputum sample from potential TB patient who is living with HIV/AIDS. 3. Conveying questionnaire screening positive patients to county level health center to get chest X-ray.	Not reported	1. The financial incentive was not given to VD on time; 2. The workload of county level health professionals were increased by having more referred patients.	1. The financial incentives for VD in finding a TB positive patient.
Li Ye, et al. [79]	2011 Shanghai Ctiy	Community TB team, including CHWs	6 months	1. Implementing publicity of tuberculosis prevention and medication safety; 2. Providing monthly door and telephone supervision; 3. Launching quarterly discussion, urging patients to use drugs, explaining the national drug relief policy, and monitoring adverse drug reactions	Not reported	1. The DOTS strategy needs to be tailored.	1. Health professional was the key to introduce TB prevention and explain other health information to patients; 2. Organizing face-to-face counselling between doctors and patients; 3. Explaining the reimbursement in detail to patients to reduce the withdrawing treatment due to low income; 4. Illustrating and explaining TB using materials that easy to understand; 5. Protecting privacy of patient
Wu Bo, et al. [80]	Not reportedChongqin City	VDs	6 months	One-to-one direct educate the residence in the community on TB	Not reported	1. The educational level of residents in rural areas was low. Traditional approaches of health education, using public board, newspaper, magazines, was not effective. 2. Elderly people had less engagement in TB health educational activities;	1. Tailored health education approach is suitable for local economic and educational level; 2. Designing and implementing appropriate approaches for different groups of residents
Chen Xi, et al. [83]	2009 Hunan Province	VDs	5 months	1. Door-to-door visit for AIDS prevention knowledge education and education materials and condoms distribution before the migrant workers leave the village; 2. Follow-up education and behavior intervention by telephone and text message after migrant workers left.	Two trainings during 2007–2008 for 317 VDs in 5 counties/villages. Training includes the basic knowledge of AIDS, methods of AIDS prevention, identification of common clinical manifestations of AIDS, consultation and referral services for suspected infected persons, etc.	1. It was difficult to manage migrant workers who often change their jobs; 2. The quality of VDs are difference; 3. There are serious discrimination against HIV infected persons in rural areas; 4. The time of returning home for migrant workers was short; 5. Residency in rural areas are scattered; 6. VDs was lack of communication skills and worried about the discrimination; 7. There were some traditional beliefs in rural areas impede the HIV education; 8.There is no specific regulation on VDs responsibility in participating the HIV prevention and control; 10. The subsidy for VDs was not in time	1. Training changed the VDs’ perspectives towards HIV/AIDS; 2. Providing appropriate subsidies to VDs since the VD services were incorporate into current public health services system; 3. Having support from policy and administrative.
Duan Song, et al. [84]	NA Yunnan Province	VDs and Peer Educator (volunteer)	Not reported	1. Implementing one to one education on HIV prevention with brochures; 2. Training home nursing staffs; 3. Distributing free condoms and demonstrating the use of condom; 4. Providing voluntary counseling and testing for HIV; 5. Training peer educators (volunteers); 6. Follow-up HIV patients and prescribe basic medication; 7. Offering various support to family members of HIV patients.	AIDS related training (did not find detail information in the article)	Not reported	1. Family based and community based care model; 2. Providing comprehensive services.
Xu Xuejiang, et al. [48]	2016 Chengdu City, Sichuan Province	Community Health Services Team, including CHWs	4 years	Community health services based HIV/AIDS preventions for female sex workers	Not reported	1. Intervention needed to be strengthened; 2. Only few health workers in community which were part-time and quick turnaround. 3. Lack of incentive mechanism.	1. Community health services centers are familiar with the environment and close to the target population; 2. The interventions conducted by community health services centers are more timely and are initiated more frequent.
Tobacco Control							
Li Jianping, et al. [67]	2009 Tianjin City	CHWs	2 months	1. Setting up smoking cessation clinic; 2. Promoting tobacco control in target community using brochures, posters, and board.; 3. Launching health education activities and collecting signatures for smoking cessation.	Training were instructed by expertise from the city level CDC and a tertiary hospital. The content includes smoking hazards, smoking cessation methods, smoking cessation skills, and management skills, to improve tobacco control ability of CHWs.	1. The intervention time was too short; 2. Education without other compulsory measures may be not strong enough to combat with nicotine addictive.	Suggestions from the author: 1. Developing long-term planning, extending the intervention time, and increasing the intensity of intervention; 2. Strengthening legislation on tobacco control; 3. Increasing scientific research in the field of smoking cessation.
Wu Xiaoli, et al. [75]	2014 Shanghai City	CHWs	12 months	1. Distributing smoke-free endorsement card to pregnant women; 2. Face-to-face health education; 3. Distributing intervention booklets 4. Playing tobacco control video courses for pregnant women; 5. Home visits; 6. Telephone follow-up.	Not reported	1. The effect of knowledge dissemination had reached a bottleneck due to the popular use of many social media platforms; 2.The intervention only affected the family level	1. Pregnant women were more sensitive to health; 2. Incorporating the intervention with the existing pregnancy insurance service system 3. Videos can largely reduce the cost of face-to-face demonstrating skills on refusing SHS.
Child Health and Vaccination							
Jianbin Zhang, et al. [72]	2005 Hunan Province	VDs	9 months	1. Storing HBV vaccine 2. Vaccinating newborns with HBV vaccination 3. Using auto-disable syringes to vaccinate newborns. 4. Using HB-Uniject™ to store vaccines and vaccinate for those newborns cannot covered by cold chain in remote area.	Not reported	1. Cost were increased by using HB-Uniject™ as injector	1. HBV vaccine can be stored in room temperature; 2. HB-Uniject™ is easy to use with accurate dose and time saving.
NCD related - Diabetes and/or Hypertension							
Wei Qiao et al. [57]	2014 Shanghai City	VDs	1 year	1. Provide health education regularly; instruct diabetes patients to test their daily blood glucose and blood pressure; monitor the blood glucose level remotely; give advice on diet, exercise, and lifestyle for patients.	Not reported	The clinical skills of VDs need to be improved.	1. This intervention program is in accordance with the government policy in health. 2. The remote surveillance platform solved the transportation issue for rural areas.
Junfeng Ji [58]	2015 Shandong Province	VDs	1 year	Patient follow-up at least four times every year (weight, heart rate, BMI, and asking for diabetes condition and lifestyles); complete the health profile for diabetes patients	Training for the process of follow-up a diabetes patient, lifestyle and treatment adjustment for patients who did not maintain their blood glucose well.	1. VDs are lack of knowledge for diabetes. 2. The average age of VDs is old. Multiple task and over workload for VDs. 3. Patients did not realize the serious impact of diabetes complications. 4. Economic issue for some patients.	Not reported
Cengceng Chen & Hui Li [59].	2016 Shandong Province	VDs	1 year	Patient follow-up four times a year.	Five trainings provided by the program including treatment for hypertension, essential drugs or medicine, case study, and health education skills.	1. Diagnostic and disease prevention skills need to be improved among VDs.	1. Strong bond between VDs and the local patients
Ren Hui, et al. [44]	2016 Shanghai City	Community Health Service Team, including general practitioner, community nurse, public health physician, and lay health worker(or non-medical workers?)	6 months	1. Intensive group intervention: nurses introduce self-management; general practitioners make rehabilitation plan with individual patients; 2. Follow-up: public health physicians monitor patients; non-medical workers organize group intervention and coordinate with patients.	Not reported	Not reported	1. Redesigned health delivery system based on chronic disease care model. Involving nurses, public health physicians, and lay health workers; 2. Patients felt more respects on their opinions and their decisions of the disease management;
NCD related - Cancer							
Chen Liang, et al. [45]	Shanghai City	General practitioner-led health management team, including community nurses	3 months	1. Establishing personal health record of patient; 2. Exercise guidance; 3. Nutrition intervention; 4. Sleep regulation; 5. Remission of pain; 6. Correct anemia; 7. Psychological intervention.	Training content includes basic knowledge of breast cancer and cancer fatigue, systematic assessment of cancer fatigue, mitigation methods, dietary guidance and medication knowledge, etc	1. The intervention time was too short; 2. Overload work for these CHWs; 3. Lack of human resource and funding; 4. The intervention only target at patients but not their social support system.	Not reported
Du Ling, et al. [46]	2013 Nanjing City, Jiangsu Province	Community workers and community nurses	3 months	1. Telephone calls and home visits, group health education activities organization, motivational interviews in peer support group. 2. Communicating with patients, and building the bridge between patients and physicians.	Not reported	1. Patients were very easy to be infected by negative mood of peer educator;	1. costs of voluntary peer support was low; 2. Peer educator has sympathy with patients; 3. Community workers can offer social and psychological support for patients as the extension and complement of the clinical services.
NCD related - Mental Health							
Jiang Yaqin, et al. [54]	NA Shanghai City	Neighborhood committee staff, community psychiatric doctors and volunteers	6 months	Programmed training: 1. Training of drug self-management skills; 2. Training of symptom self-monitoring skills	Not reported	Not reported	1. Programmed skill training is effective in relieving mental symptoms, improving self-knowledge and social function;
Shu Dalin, et al. [65]	2010 Hunan Province	Community Health Service Team, including CHWs	2 years	Community comprehensive intervention: 1. Health education; 2. Drug intervention; 3. Psychological intervention; 4. Life intervention; 5. Rehabilitation training; 6. Follow up and health evaluation.	Not reported	1. Lack of funding and mental health workers; 2. Huge economic burden for families with financial difficulties during the long-term intervention; 3. Patient disturbance during the intervention was difficult to solve without civil administration and public security department.	1. Community health intervention can be flexible and practical. 2. Community health intervention can largely reduce the burden of their family and the society.
NCD related - Cardiovascular diseases & Hypertension							
Guan Fei, et al. [69]	2005 Henan Province	Community General Practitioners	1 year	Hierarchical Risk factors management intervention: 1. Dissemination of health knowledge using lectures, training course, free counselling, contest, and distribute education materials; 2. Psychological assessment and counseling including phone and face-to-face counselling; 3. Full-course demonstration intervention of family health	Not reported	1. Obesity and overweight rates of body mass needed long-term intervention; 2. the intervention stage is short, and the effect of some intervention project was not obvious; 3. cardiovascular endpoints were not observed;	1. Management of the whole population, including healthy population, high risk population, and patients; 2. Educating the family members of the patients, especially those who had the right to decide the health education of patients with cardiovascular diseases was effective

In terms of the duration of these programs, a few studies lasted for 2–6 months [26–40]. The majority of the studies, including 18 NCD studies lasted more than one year, even a few years [25, 41–48]. Some others, including a family planning, a mental health, and 4 tuberculosis-related studies evaluated the on-going programs [21, 25, 49–52].

Most of the studies (18 studies) were related to NCDs covering five major areas of diabetes and/or hypertension [28, 29, 43, 44, 53–59], cancer (h [45, 46]), mental health [32, 33, 52, 60–65], cardiovascular diseases [66–69]; and NCD health education [47, 70]. Ten articles were related to reproductive health, including family planning, prenatal care, and postnatal care. Besides family planning and maternal health, other services provided by CHWs includes managing patient with infectious disease like tuberculosis (TB) (10 studies), HIV (3 studies), child health (one study provided early childhood development consulting [61] while another study provided counseling for children second-hand smoking exposure [62]), immunization (4 studies [70–72]), and others (one study focused on shallow anterior chamber screening [73], one study conducted verbal autopsy [74], and two studies for tobacco control [36, 75]).

The terms used to define CHWs varied in different studies. Most of the studies used village doctors (VDs) or community health workers as CHWs (*n* = 42). In family planning and maternal health care particularly, traditional birth attendances (TBAs) (*n* = 2), village/grassroots maternal health care workers (*n* = 3), traditional village midwives (*n* = 1), family planning workers/staff (n = 4), outreach providers (n = 1), and village nurses (*n* = 8) were also used. In those NCD studies, other terms for CHWs include lay family health promoters (n = 2), lay health supporters (n = 1), health coach (n = 1), non-professional health workers (n = 1). In Chinese literature, particularly, community nurses and CHWs were referred in a health management team (*n* = 5).

### Public health services that CHWs provided

Public health services provided by CHWs were various depending on the types of studies and programs. In most of the studies, CHWs served as program recruiter and health aides providing health education and assisting patient management.

#### NCD related services

In all the identified NCD-related programs CHWs mostly assisted clinicians to promote screening for major NCDs. In some studies, they provided lifestyle modification supports via counseling and educational sessions among NCD patients and people at risk [28, 30, 46, 47, 54, 56, 57, 65–68, 70]. The content of such counseling support included healthy diet, physical activities, mental health self-management, smoking cessation, salt intake reduction, and practical approaches to prevent unhealthy behaviors. CHWs also helped in monitoring patients’ medication adherence in regular follow-ups, reporting side-effects, and referring severe cases to the higher level medical facilities [29, 44, 52, 58, 59, 65–68]. In addition, several studies reported that well-trained CHWs with sufficient technical support could distribute mental health medicines [52, 61, 65], measure blood pressure, directly conduct early detections for CVDs or diabetes, as well as prescribe blood pressure lowering drugs and aspirin for CVDs [28, 43, 56, 66–68].

#### Reproductive health

Among the studies that focused on reproductive health, consisting of family planning and maternal health, CHWs mostly provided outreach home visit services [23, 26, 27, 41, 76–78]. CHWs also provided health education to pregnant women and their companions on specific topics, including prevention and control of sexually transmitted infections [79], maternal newborn danger sign recognition [41, 76], antepartum and postpartum care seeking and nutrition [27, 41, 76, 78, 80], and breast feeding [76]. They were also in charge of distributing contraceptives, managing and supervising contraceptives as family-planning workers. Other services provided by CHWs included distribution of nutrient supplements for pregnant women [80], conducting mini-survey [80], monitoring compliance of supplements taking [80], and offering general or specific health promotion counseling [23, 25, 27, 78, 81]. Only one study mentioned that CHWs attended births [41].

#### Infectious diseases (tuberculosis and HIV)

Guided by Directly Observed Treatment, Short Course (DOTS) strategy, the major responsibility of CHWs was to provide direct observations for smear-positive TB patients in most of the studies [21, 24, 37–39, 49, 50, 82]. Meanwhile, they detected new TB cases, followed up TB patients, referred patients to higher level TB dispensaries and designated sputum examination centers, as well as conducted relevant surveys or collected relevant data for research teams [51, 82, 83].

Four studies [48, 78, 83, 84], all in Chinese literature, reported using VDs to support HIV prevention. In one study, VDs screened potential TB patients who were living with HIV [78]. In other three studies, VDs and volunteers provided health education on HIV prevention for migrant workers [83], HIV patients and their family [84], and female sex workers [48].

#### Child health and Vaccination

CHWs provided early childhood development consulting in the two child-health-related studies [61]. In the immunization-related study, CHWs monitored children’s immunization status and reminded their caregivers to get the child vaccinated [22]. In other studies, village-based health workers administered immunization shots using auto-disposable syringe and vaccine storage in rural areas to ensure the timely immunization for Hepatitis B birth-dose [71, 72].

#### Tobacco control and other services

Two of the tobacco control studies targeted specific population. In those studies, CHWs were responsible for individualized counseling to parents on second hand-smoking exposure to children [62] and to family members of pregnant women on passive smoke exposure to pregnant women [75]. In another study, CHWs provided general tobacco control intervention in the community [67]. In two studies, CHWs played other roles like screening shallow anterior chamber or conducting verbal autopsy based on the research program [73, 74].

### Training received by CHWs

#### Training content

Among all the identified articles, 38 studies indicated the training of CHWs and 34 of these studies reported details of the training content. The content of CHWs’ training was relevant to the services that they would provide. For example, in maternal health-related studies, CHWs usually received training on basic knowledge about maternal health, conducted prenatal visits, and identified danger signs [41]. While for the CHWs who conducted or assisted in NCDs screenings, generally acquired knowledge on the disease-related risk behaviors, how to detect suspicious cases [66, 68], how to screen [67], and the meaning of the positive test [45]. The level of training received by CHWs differed across studies. For example, in a study conducted in Guangxi, the training for TBAs is focused on care during childbirth and referral skills while the training for trained birth attendances (TBAs) included additional midwifery training and conducting of at least 30 independent deliveries under an obstetrician’s supervision [77]. CHWs also received other types of training including health education communication skills [23], computer skills [79], mobile phone app use [22], TB/HIV control and management [40, 82], and verbal autopsy interview skills [74].

#### Types of training

Seventeen articles described the types of training for CHWs. Most trainings were given by teachers or experts through lectures. In-class and group discussions as well as role-plays were used frequently in CHWs trainings [23, 32, 35, 76]. Some NCD studies also used web-based trainings combined with video, picture, and text for CHWs [28, 43, 46, 64]. Two studies mentioned reflection-action-assessment cycle methods and case review [35, 42]. Besides formal training, one article also delivered desk calendars with TB information and control policy to village doctors, village leaders, and patients [83].

### Challenges

Forty seven articles indicated various challenges in the CHWs led projects. The common barriers are: lack of transportation, lack of official support, poor capacity of CHWs, lack of training for CHWs, incentives for CHWs, and establishing and maintaining the relationship between CHWs and target population in the community.

#### Transportation

Four articles mentioned the challenges in transportation to reach the community [24, 49, 77, 82]. In remote areas, institution-based delivery was hard to perform without proper logistic support [77]. Village doctors also reported that it was hard to launch DOTS with an inconvenient transportation system [24, 49, 82]. Additionally, one literature mentioned that the residency of target population (i.e. migrant workers) in rural areas are scattered [83].

#### Official support

Official support includes the financial and policy supports from government and the understanding from local stakeholders. In China, policy is a guide for family-planning workers and other government-funded programs. In the study of Tu et al. (2004), family-planning workers were unsure of the need for the government agency providing reproductive health education to unmarried young people [25]. Another study discussed the concerns from local leaders about the utility and appropriateness for involving village health workers with little formal education. These concerns affected the long-term commitment of key trainers to provide training or some CHWs to receive training [42]. One study emphasized the need of government support both on funding and regulations [67]. Another two studies pointed out the need to involve stakeholders such as family planning, civil administration, women’s federation, administration of justice, and public security department [65, 81].

#### Quantity and quality of CHWs

CHWs usually have heavy workload by providing both their assigned routine duties and public health services at the same time. One of the articles used “shortage of hands” to indicate this barrier [24], reflecting the workloads and demands of their work. Additionally, the village doctors who were already busy in providing general primary healthcare services were reluctant to add extra NCD related tasks on their agenda [43, 52, 61]. On the other hand, some CHWs lacked adequate knowledge and capability to meet the demand of their assigned work [40, 47, 51, 57–59]. Other studies also indicated CHW’s lack of specific skills as barriers [24, 42, 51, 58, 59, 70]. One study pointed out that the average age for VDs are getting older [58].

#### Training of CHWs

The training received by CHWs was diverse and related to various education levels of CHWs, different learning needs, too many trainees, and trainers’ unfamiliarity with the work of CHWs, especially in programs that used technological support [23, 24, 41, 42, 70, 74, 77]. Less than 40% (8 out of 22) of the Chinese literature reported detailed training for CHWs. Most of these trainings were provided as lectures and evaluated by tests. None of the studies discussed the challenges in training CHWs.

#### Incentives for CHWs

Varieties of motivational factors to engage CHW in public health service delivery was described across studies. Ten articles emphasized inadequate financial incentives for CHWs [23, 24, 30, 34, 37, 40, 49, 65, 82, 83]. Different issues of financial incentives include the shortage of funding [30, 65, 83], lack of subsidies [23], specific allowance/incentives did not reach to CHWs [24, 78, 83], and no additional financial reward [61]. Another article reported lack of recertification mechanism as barrier for motivating CHWs to attend training and learn more medical knowledge [79].

#### Maintaining relationship between CHWs and target population

The main barrier in maintaining the relationship between CHWs and target population is the mobilization of the target population [34, 40, 70]. Non-permanent job status of CHWs was another barrier to build rapport [41]. Population mobility was a barrier to maintain relationship in programs of TB, HIV and immunization [34, 70]. A study mentioned the difficulties to involve elderly people in the intervention [80].

### Facilitating factors

#### Official support

In China, official support is crucial for CHWs-led health program. Several studies emphasized the official support from government and clinic as a facilitator in their studies [40–42, 78]. Similarly, Wei et al. (2008) underlined the importance of the leading role of the local policy maker while making changes in policy and practice in primary health care [82]. The nationwide program used a top-down approach with specific task assignments to CHWs, which was effective in TB control and management [21].

#### Integration of CHWs programs within the existing health systems

Although CHWs were integrated in the existing health system, a well-designed health intervention program which could be fitted into the current system as the routine task for CHWs was identified as one of the facilitators. A tobacco control study mentioned one of the facilitating factors as incorporating the intervention with the existing pregnancy insurance services system [75]. Edward and Roelofs (2006) emphasized the good fit between core project elements and the existing health system when designing their health intervention project. Deep engagement of local partners was a good approach to ensure effective implementation of the CHW-led program [42]. Jiang et al. (2016) discussed the need for sufficient and comprehensive preparation within the health system in order to develop a well-designed intervention program [77]. These preparations include training of health human resources (i.e. CHWs), building infrastructure, improving services quality, and establishing referral system with quality referral center.

#### Relationship between CHWs and residents

The good relationship between the CHWs and residents is an important facilitating factor. The team-based model is becoming more common [29–31, 33, 38, 48, 65]. The benefit of involving the CHWs in the multidisciplinary health management team is that they can act as a bridge between the team and patients [59]. Because the CHWs always work closely with the community, they can provide intervention conveniently and frequently [48]. Moreover, it is easier for the CHWs to educate the family members of the patient compared to physicians [69, 85].

#### Financial support

Four articles mentioned financial support as a facilitator for CHWs engagement in healthcare delivery [24, 37, 40, 77]. Financial compensation for CHWs was provided by local health institutions based on the services that they provided (i.e. the number of pregnant woman escorted to the health institute) [77]. They could also receive additional payment if they provided other services, including prenatal/postpartum examination referral to a health facility [77]. One study suggested non-monetary incentives like food, uniform or public praise as substitutes to cash allowance [24]. Few studies suggested that performance-based incentives were effective in increasing CHWs’ job motivation and improving their work performance [43, 46, 67, 68].

#### Technology support

Overall, ten studies used the website or mobile phone applications to facilitate CHWs-led programs. Seven of them were NCD-related [24, 46, 55, 57, 64, 66, 68] and only three studies were related to general service provision [22, 74, 79]. One study used the website as a training method to provide specific training for village doctors [79]. Another study used a mobile phone-based application to support health management system in improving immunization management and tracking by CHWs [22]. Zhang et al. used mobile phone-based application to facilitate decision support system for verbal autopsy interviews by CHWs [74].

## Discussion

To our knowledge, this is the first systematic review that provides a critical appraisal of health programs delivered by CHWs in China during the last two decades. We found that, overall, CHWs provided varieties of services that were relevant to the national policy for basic public health services and the national priority public health programs. We found that family planning and reproductive health services were more frequently being studied and reported in the review. It could be partially explained by the family planning policy initiated in 1983 that required the National/State Ministry of Health and Population and Family Planning Commission working closely with local CHWs and village doctors [86]. Similarly, CHWs were also engaged in the implementation of national programs of DOTS [87] and Expanded Program on Immunization (EPI) which was initiated in 2008 [88]. These engagement of CHWs in major national programs suggests that the government realized the importance of CHWs in promoting public health programs underscoring the potential for their integration into the existing primary healthcare systems. In programs where CHWs were engaged to work in specific project with funding for limited duration [62], extra efforts need to be made to keep these CHWs engaged in the same community based programs, if the program proven to be effective.

We found that there was no consistency in terms of duration and intensity of the training received by CHWs in the studies reviewed since most of the trainings were on-the-job training (i.e. specific trainings were given in relation to specific tasks). While this is understandable that the training was customized to the needs of the specific program, a basic training on core competencies would improve the quality of the service delivery and the overall skills of CHWs. Earlier studies reported several barriers in relation to the training of CHWs, including relatively low educational levels of CHWs [42], too many trainees while a few available trainers [23], and technology usage in the training for elderly CHWs [70]. In the current study, we also identified similar barriers for training CHWs, which underscore the importance of considering these in the planning of any training for CHWs.

One of the crucial factors for CHWs to implement programs effectively was the level of official support received from the national and local government as well as other stakeholders. Essentially, the support from the government could be both a barrier and a facilitator. In our review, studies found that ambiguous policies and perspectives from local leaders could impede CHWs implementing health intervention programs. However, if the health intervention program could receive the official support from government and health centers, these supports would be great facilitators [41, 42]. In China, using a top-down approach turned out to be effective for many nationwide health programs (e.g. Patriotic Sanitation Campaign started from 1952). With a specific policy or working guide, CHWs would have a proper perspective of the provision of primary health services. Moreover, the government is responsible for the construction of infrastructure including improving transportation system and building community health centers and village clinics. Road accessibility was one of the basic requirement for proper logistic support, particularly in rural areas and remote villages [77]. The convenient transportation system was needed for CHWs to effectively launch DOTS strategy, either for patients coming to the CHW’s office or for the CHW’s visit to patients’ home [24, 49, 82].

In the studies reviewed, we identified several factors that were relevant in keeping CHWs motivated to their job. These included reduced workload, financial and non-financial incentives, regulation and continuous education, integration of CHWs in the current health system, and the job satisfaction of CHWs. These factors are consistent with previous findings in low- and middle- income countries [89]. In earlier studies, village doctors were most dissatisfied with their pay and the amount of work, as well as promotion and work conditions [90, 91]. Few other studies also reported that low salary and lack of financial incentives were substantial barriers for motivating CHWs [24, 34, 83]. Thus, appropriate financial incentives system is valuable for the whole health-care system to retain CHWs in their existing jobs [89].

Information and communication technologies or mHealth (i.e. internet, mobile phone applications) was frequently used in recent years by the researchers in CHWs delivered programs. It could be an effective approach to improve the consistency and efficiency of health services delivery by CHWs [92, 93]. Although only three studies used website and mobile phone applications in general service provision, the outcomes were promising [22, 74, 79]. For example, in the study of Chen et al. (2016), the use of Expanded Program on Immunization (EPI) application improved the local full vaccination coverage and working efficiency of CHWs [22]. This finding is consistent with a previous systematic review on CHWs and mobile technology, which indicated that new technologies could assist CHWs to improve the quality of providing health services, the efficiency of health intervention, and capacity for program monitoring [92]. One of the barriers mentioned in reviewed studies was the elderly CHWs as they might not familiar with smart phone applications or not willing to learn about new technology. Future studies could focus on developing user-friendly applications and should plan to provide multiple training for elderly CHWs.

To ensure the sustainability of the health intervention program, China benefits form its institutionalization of CHWs as part of the primary healthcare providers (VDs and community nurses). This is similar to the program in Brazil, the Brazilian Family Health Programmer, which integrated CHWs into its health services and institutionalized community health committees to ensure the sustainability of health care delivery [94]. Both China and Brazil also benefit from the multidisciplinary health care team in the primary care setting, which may include CHWs, psychiatrists, general practitioners, nutritionists, public health specialist, and others.

Unlike the high-income countries, where CHWs mainly focus on marginalized population [95], Chinese CHWs provides health care services for all members in the community, based on the programs to which they are assigned. Therefore, the potential to generalize and expand the CHWs-led programs in China is great and should be explored further.

## Public health implications

The findings of this review have several implications.

### Motivation of CHWs and health-care reform

The motivation of CHWs to engage in public health service delivery was influenced by the whole range of health sector reform [96]. In 2009, a new health-care reform was launched aiming to achieve universal health coverage. This health-care reform dramatically reduced the income of village doctors by canceling the drug mark-ups which was the primary source of income for village doctors for more than a decade [97, 98]. Realizing the irreplaceable role of CHWs and the lack of financial incentives for primary health services, Chinese Ministry of Health issued the National Basic Public Health Service Standards as a guideline for primary health services in 2011. The Ministry of Health also increased the compensation for primary health services per capita in recent years. However, the compensation was still insufficient and sometimes even did not reach to the CHWs [97]. In 2015, the average compensation for basic public health services per person increased from 35 to 40 RMB (1US$ = 6.90RMB). Measures need to be taken so that the increased compensation would reach to CHWs to motivate them in the delivery of primary health services. However, the effectiveness of this new policy hasn’t been evaluated.

In terms of the incentive mechanism, Tao et al. (2013) suggested that performance-based incentive could be an effective approach to improve the performance of CHWs in tuberculosis DOTS strategy [24]. However, a systematic review by Kok et al. (2015) indicated that this approach could sometimes lead to ignore the unpaid task of CHWs’ daily work [99]. Although pay-for-performance has become popular in recent years to initially improve the performance of health professionals while controlling healthcare expenditure, policy makers should carefully design the payment system to reach their initial goals in China [100]. To provide more evidence for policy makers, future research studies could focus on exploring and evaluating salary and incentive mechanisms which could effectively motivate CHWs and improve health care service quality as well as the cost-effectiveness of the intervention.

### Ensure long-term commitment of CHWs

In China, CHWs were part of the primary health care system, whose salary were covered by the government. However, engaging these CHWs in primary health care with long-term commitment may be not easy. The long-term commitment of CHWs in the primary health-care system is a prerequisite for sustainable health intervention program engaging CHWs. This long-term commitment could greatly be influenced by regulation and continuing education for CHWs. These two factors could help village doctors building long-term perspectives of their career and motivate them to keep learning and practicing in primary health care.

A regulation for CHWs issued in 2003 called the *Regulation on the Administration of the Practice of Rural Doctor*, which took effect in 2004 [101]. The registration system in this regulation requires village doctors to be trained by local health department at the county level. After the training, village doctors must pass the license examination to be qualified. The challenge to execute the regulation is the low educational level of the current village doctor. Although the national continuing medical education system requires professional physicians and nurses in the township or higher level to attend training and take an exam, it does not have corresponding requirements for village doctors. Other than the 2005’s regulation, there are neither relevant regulations nor financial support for continuing medical education and promotion for village doctors.

Since there are no systematic and professional continuing education for CHWs regulated by the government, these trainings were mainly based on the different needs of the health intervention programs. However, this should be noted that the training process for CHWs was rarely described in reviewed studies while few earlier studies had ever explored the effective training for CHWs. The training for CHWs could be a valuable reference when designing similar primary health care intervention program in future studies as well as offering evidence for policy makers to designing continuing medical education for CHWs. Thus, researchers should highlight the importance of CHWs training when designing health intervention programs involving CHWs in the future.

## Limitations

A few limitations of this review should be noted. First, because many terms are used to describe CHWs and front-line public health workers, it is possible that we were not able to extract all relevant articles in the existing literature. However, to avoid this, we conducted a systematic electronic search using a comprehensive list of Medical Subject Headings terms as well as similar keywords, such as village doctors or lay health worker, after a consultation with a community health advocate in China and a trained health science librarian. Second, we included both English and Chinese literature. However, most Chinese literature did not discuss the challenges and facilitating factors of their intervention program. Thus, the challenges and facilitating factors were mainly extracted from the English literature. Third, most of the studies reviewed were conducted in rural areas in China. The ethnic and cultural diversity across China limits the generalizability of the findings to all the provinces and cities [25]. Fourth, the findings we summarized are based on the reports in the published paper. No attempts were made to assess the quality of the published reports or to validate the findings or conclusions of the reported studies. Finally, we did not take effort to identify grey literatures and might have missed studies as a result. Therefore, the findings of the current paper need to be extrapolated considering these limitations.

## Conclusion

Involving CHWs in the delivery of public health programs has a long history in China. We found that a significant amount of research was conducted in China that involved CHWs. This review has provided insights into the pattern of public health services provided by CHWs in China and summarized potential barriers and facilitating factors. This will allow policy makers and other stakeholders to determine how to engage CHWs to address the growing need for public health services and community based care for diverse healthcare needs. As China is going through healthcare reform, incorporating CHWs as a member of the primary healthcare workforce within the healthcare delivery systems, with enhanced training and continuing training, will motivate CHWs to engage and deliver high standard community-based healthcare services.

## Abbreviations

CHW: Community Health Worker
DOTS: Directly Observed Treatment, Short Course
EPI: Expanded Program on Immunization
NCDs: Non-communicable diseases
RCT: randomized control trial
TB: tuberculosis
TrBAs: Trained birth attendances
VDs: village doctors
WHO: World Health Organization
